# Non-invasive genetic monitoring involving citizen science enables reconstruction of current pack dynamics in a re-establishing wolf population

**DOI:** 10.1186/s12898-017-0154-8

**Published:** 2017-12-19

**Authors:** Hanna Granroth-Wilding, Craig Primmer, Meri Lindqvist, Jenni Poutanen, Olaf Thalmann, Jouni Aspi, Jenni Harmoinen, Ilpo Kojola, Toni Laaksonen

**Affiliations:** 1 Department of Biology, University of Turku, Turku, Finland; 2 Present Address: Ecology & Evolution Division, Department of Biosciences, University of Helsinki, Helsinki, Finland; 3 Present Address: Department of Biosciences & Institute of Biotechnology, University of Helsinki, Helsinki, Finland; 4 Present Address: Department of Pediatric Gastroenterology and Metabolic Diseases, Poznan University of Medical Sciences, Poznan, Poland; 5 Ecology and Genetics Research Unit, University of Oulu, Oulu, Finland; 6 Natural Resources Institute (Luke), Rovaniemi, Finland

**Keywords:** Evidence-based conservation, Human–wildlife conflict, Predator, Stakeholder involvement, Wildlife management

## Abstract

**Background:**

Carnivores are re-establishing in many human-populated areas, where their presence is often contentious. Reaching consensus on management decisions is often hampered by a dispute over the size of the local carnivore population. Understanding the reproductive dynamics and individual movements of the carnivores can provide support for management decisions, but individual-level information can be difficult to obtain from elusive, wide-ranging species. Non-invasive genetic sampling can yield such information, but makes subsequent reconstruction of population history challenging due to incomplete population coverage and error-prone data. Here, we combine a collaborative, volunteer-based sampling scheme with Bayesian pedigree reconstruction to describe the pack dynamics of an establishing grey wolf (*Canis lupus*) population in south-west Finland, where wolf breeding was recorded in 2006 for the first time in over a century.

**Results:**

Using DNA extracted mainly from faeces collected since 2008, we identified 81 individual wolves and assigned credible full parentages to 70 of these and partial parentages to a further 9, revealing 7 breeding pairs. Individuals used a range of strategies to obtain breeding opportunities, including dispersal to established or new packs, long-distance migration and inheriting breeding roles. Gene flow occurred between all packs but inbreeding events were rare.

**Conclusions:**

These findings demonstrate that characterizing ongoing pack dynamics can provide detailed, locally-relevant insight into the ecology of contentious species such as the wolf. Involving various stakeholders in data collection makes these results more likely to be accepted as unbiased and hence reliable grounds for management decisions.

**Electronic supplementary material:**

The online version of this article (10.1186/s12898-017-0154-8) contains supplementary material, which is available to authorized users.

## Background

Large carnivores are important for ecosystem sustainability and diversity, but in many places they have declined drastically due to anthropogenic pressure [1, 2]. Carnivores frequently come into conflict with human interests as they prey on domestic and game animals and are often perceived as a threat to human safety [2–4]. As a consequence, many large carnivores have been eradicated from wide geographic ranges to date. However, strict conservation measures have recently enabled their return in some areas, including Europe [5] and North America [6]. Re-establishment of such emotive species to urban and agricultural landscapes often triggers public concern as local people are no longer accustomed to the presence of predators. Indeed, a better understanding of the re-establishing species’ biology has been shown to be essential to minimizing conflict and fostering sustainable coexistence of humans and wild carnivores [7, 8].

One of the most controversial and conflict-prone carnivores is the grey wolf (*Canis lupus*). Following near extirpation from most of Europe and North America, the species is now resettling in some parts of its previous home ranges [5, 9]. One such area is southwestern Finland, which wolves have recently recolonized as breeders after an absence of ca. 120 years [10, 11]. This area has a tragic history with wolves that affects the social environment for their conservation and management: in 1879–1882, > 20 children were killed in putative wolf attacks and local wolves were subsequently eradicated by professional hunters [12]. Rebounding started in 2006 when the first family pack was confirmed [10]. These historical events have contributed to a pronounced conflict over the re-establishment of wolves between enforced, top–down conservation aims (e.g. strict protection under the European Union’s Habitats Directive) and local inhabitants’ concerns for the safety of their families, livestock and pets [8, 11, 13]. This cultural context, together with incidences of wolf sightings close to human settlements [14], has provoked strong calls for the complete eradication of wolves from the area [8, 11].

A key element in conflicts over the management of carnivores is typically a lack of information or disagreement regarding basic biological facts related to the carnivores. In the case of wolves in SW Finland, there have been several debates about the number of wolf packs and individuals, in which the opinions of different stakeholders vary and some local residents mistrust the official estimates [8]. Reliable identification of individual wolves in this area could help to resolve the dispute over numbers of wolves, providing an unbiased basis for conservation and management, but this is not possible with the common census approach of track-based counts. To this end, genetic methods using non-invasively collected samples that allow individual identification but do not require capturing, handling or direct physical contact with the animals have recently provided a useful tool for studying the population size and structure of rare and elusive species [15–18] including various wolf populations [19–21]. However, few genetic monitoring studies have thus far taken advantage of the insight that such data can provide on management-relevant reproductive dynamics [e.g. 21, 22]. In a re-establishing population, robust characterization of relatedness patterns allows (1) reconstruction of the colonisation history at the individual level, (2) identifying breeding pairs, and (3) inferring likely future breeding events. Understanding these dynamics provides a basis for targeted management actions [16], both immediate—e.g. identifying problematic groups or individuals for control—and anticipatory, such as predicting changes in pack configurations based on past responses to similar pressures. Investigating these patterns of population change is particularly valuable in a recently-established and currently expanding population such as the SW Finland wolves, where pack development can be followed in almost real time and predictions of change tested in the context of e.g. harvesting of identified breeding individuals.

Particularly when data collection is based on citizen science—in which interested non-experts contribute to data collection—non-invasive genetic sampling provides an efficient alternative to deliver detailed monitoring of local individuals that does not rely on field observations of these elusive, wide-ranging animals and at the same time enables inclusion of different stakeholders in knowledge generation. However, non-invasive sampling also poses challenges for conventional genetic pedigree reconstruction techniques [23–26]. The genetic material can be of poor quality and prone to genotyping errors, and many parents of identified individuals may not be sampled but without a priori knowledge of which or how many individuals. Further, individuals may be both parents and/or offspring, but there is little prior cohort information to constrain these relationships. Pedigree reconstruction in a Bayesian framework provides a versatile tool to overcome these constraints in a single analytical framework, reducing potential bias caused by assumptions around unknown variables and allowing phenotypic data to be considered together with genetic data in estimating parentages [23, 25, 27].

We used a combination of non-invasive and opportunistic samples to obtain genetic data from which to characterize the relatedness structure of the re-establishing wolf population in SW Finland in a Bayesian framework. To enhance the social acceptance of our outcomes in this contentious debate, we employed a collaborative sampling framework that is primarily based on a joint effort between local people, wildlife administration and researchers. Thus, stakeholders representing a range of interests contributed to knowledge generation, with a view to decreasing conflict over knowledge ownership and bias. Using an established genotyping panel of 17 microsatellite markers [28], we first identified 81 genetically unique individuals represented in the samples. We then reconstructed a multi-generational pedigree to determine reproductive pairs, identify family packs and characterize their habitat areas. Lastly, supplementing the family structure with temporally explicit observational data, we constructed a population history of wolf re-colonisation in this human-populated area. These data are already being used as a basis for local population management and the entire monitoring framework is being expanded over a larger area.

## Methods

### Study area

**Fig. 1:**
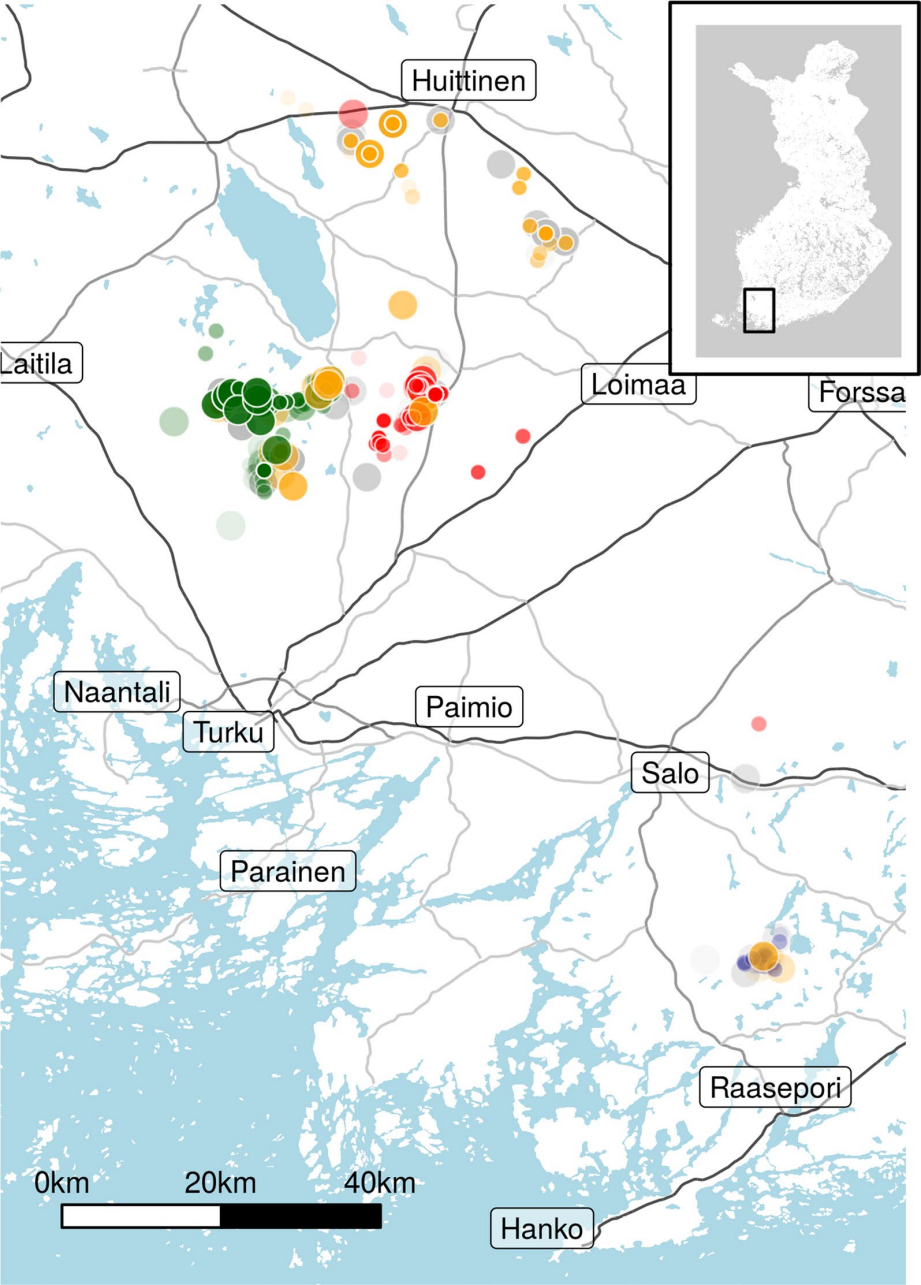
The study area in SW Finland, with the location of all samples collected, illustrating the location of pack territories. Samples are colour-coded according to their natal pack. The larger symbols denote known breeders. Individuals of unknown/uncertain parentage are shown in grey, and more recent samples are shown in stronger colours. The inset shows the whole country with the study area outlined

The study was carried out in southwestern Finland in the regions Varsinais-Suomi, Satakunta and Uusimaa (Fig. 1). Wolves were extirpated from the area in the 1880s and only returned in 2005, when a pair was formed; since 2006, wolves or tracks have been observed continually in the area and reproduction is thought to have occurred annually [10 and unpublished data]. In recent years, wolf densities in this part of Finland have been as high as in the east of the country, close to the Russian border, where wolves re-established more than a decade earlier, in the 1990s [10]. Although separated by several hundred kilometres, these two populations are not isolated as this distance is within the established dispersal range of grey wolves and the land between the two populations is predominantly forested and hence suitable wolf habitat [29, 30]. Nevertheless, the population in SW Finland represents a pool of potential mates distinct from the eastern population, as there have been only few reproductive attempts in the area between southwest and eastern Finland [10].

### Sample collection

This study used genetic data from non-invasive samples collected in 2013–2016 supplemented with genetic information available from wolves that were killed or trapped in the area in 2008–2015. Our main source material for DNA extraction was non-invasively collected fecal samples, which are relatively easy to find either by following wolf tracks, especially during winter when snow-cover makes tracks more visible, or near wolf kill sites or alongside forest roads, which wolves use when moving across the territory [31, 32]. Sample collection was initially organized and performed in collaboration with local wildlife agencies in the study area (Varsinais-Suomen and Satakunnan Riistakeskus, the Wildlife Agencies in Varsinais-Suomi and Satakunta), the Regional Wildlife Council (alueellinen riistaneuvosto) of Varsinais-Suomi, and the Natural Resources Institute Finland (Luke). Potential collaborators were invited to two meetings, and a voluntary scheme was established to opportunistically collect fecal samples. Sample collectors were mainly voluntary large carnivore personnel (hunters) that have been trained to identify wolf tracks and signs [see 10], with additional samples collected by other local hunters and nature enthusiasts that make recreational use of the area.

Sample collectors were provided with instructions and collection kits to ensure consistency in the sampling protocol and associated data. Whole fecal lobes were collected using a plastic bag and the following information was recorded: name and e-mail address of the collector, date, coordinates of the sampling location or exact written description about the sampling site, an estimate of how many days the sample may have been in the field and how many tracks were seen around the sample (if applicable). Additional information was often added by sample collectors, for example to note uncertainty that the sample was produced by a wolf. To minimize DNA degradation, samples were frozen upon collection and subsequently transported in cool bags to the laboratory, where they were stored at − 20 °C. In order to motivate sample providers, each participant received frequent feedback about the progress of the respective samples. Additionally, two progress reports were sent during and at the end of the sample collection season. From summer 2014 to spring 2016, new samples were collected by Natural Resources Institute Finland (Luke) assisted by the volunteers; the sampling scheme was not however advertised as widely as it was in 2013–2014 due to funding uncertainty. In total, this collection protocol yielded 516 scat samples, 368 of which yielded at least partial genotypes, covering adults and litters produced in the breeding seasons 2013–2015.

In addition to these non-invasive fecal samples, we also used 17 non-invasive secretion/tissue samples (urine, blood, saliva swabs from kills and hair) collected from the surface of the snow in the winters of 2014 and 2015 and 16 tissue samples, contributed by EVIRA (Finnish Food Safety Authority) and the Natural Resources Institute in 2014, from carcasses of wolves that had died of various causes (mange, car accident, legal shootings). In total, the study period yielded 394 genotype determinations sufficiently complete to be assigned to an individual, of which the majority (368; 93%), were generated from scats (Table 1). Finally, to obtain information on individuals historically present in the area, we included genotypes (N = 18) from a small number of older fecal samples (N = 32) collected in the study area between 2008 and 2012 by nature enthusiasts. We also used genotypes of 10 individuals from a nationwide dataset of individual wolf genotypes [28 and unpublished], generated from tissue or saliva samples from animals that died (N = 7) or were captured alive (N = 3) in the study area during 2008–2012 (Table 1). In addition, reference samples from domestic dogs of multiple breeds were collected as buccal swabs from 77 dogs in 2014–15 by a veterinarian at Univet clinic, Turku, and extracted and genotyped using the same protocol as for wolf tissue samples.

**Table 1:** Sample sizes at different stages of the study protocol. DNA amplification was attempted on all samples collected during the study period (2013–2015); the number of genotypes obtained and used in the analysis is lower as many samples did not yield a sufficient number of markers to assign the sample to an individual

Sample type	Samples	Genotypes obtained
Scats, study period	516	368
Tissue/secretions, study period	33	26
Scats, before study period	32	18
Tissue secretions, before study period	10	10

Samples from before the study period (2008–2012) were obtained from other contributors as genotypes

### Genotyping

Samples from a range of sources (tissue, feces, hair, urine and secretion samples) were processed as a part of ongoing monitoring procedures over several years, and genotyping was conducted by several different researchers. Therefore, the DNA extractions and genotyping protocols were modified during the course of the research. Below, a summary of these procedures is provided; full details can be found in Additional file 1: Appendix 1.

DNA was extracted using commercially available kits with minor modifications of the manufacturer’s protocols. The kit and protocol varied depending on the DNA source material (details in Additional file 1: Appendix 1). Negative controls were added in all extraction batches where samples were expected to have low quality or quantity DNA. The quantity and quality of all extracted DNA were quantified using a NanoDrop ND-1000 spectrophotometer.

All samples were genotyped at 17 established canid microsatellite loci (Additional file 1: Table S1) [33, 34] that have been previously used in wolf population genetic studies in Finland [28]. Separate amplification protocols were used for tissue and other sample types.

Amplification was conducted in 3–5 multiplex PCRs depending on the DNA source (Additional file 1: Appendix 1) in 10–12 µl volumes containing 1–3 µl template DNA, 1 × Qiagen multiplex PCR master mix (QMP) and 0.1–0.4 µM of each primer (Additional file 1: Table S1). The thermal cycling program was: initial denaturation at 95 °C for 15 min, 35–40 amplification cycles of denaturation at 94 °C for 30 s, annealing at 60 °C for 90 s and extension at 72 °C for 60 s, and a final extension step at 60 °C for 10–30 min.

Individual multi-locus genotypes were generated using fragment analysis of the pooled fluorescently labeled PCR products. The allele sizes of the microsatellite markers were determined by capillary electrophoresis using an ABI PRISM 3130xl Genetic Analyzer-sequencer (Applied Biosystems) and individual genotypes were called using the genotyping program *GeneMarker* v.2.2.0 (Softgenetics, Inc.). After an automated initial allele call, all alleles were manually checked and confirmed before a final genotype was assigned.

Genotyping was carried out a number of times on each sample, as recommended to ensure reliability of genotypes when analyzing very dilute and/or degraded DNA such as that extracted from feces [35–37]. Each sample was first amplified in three independent replicates; at each locus, heterozygous genotypes were considered reliable if both alleles were observed in at least two replicates and for homozygous genotypes if the same genotype was observed in all three [38, 39]. If any locus did not meet these requirements, a second series of three replicates was performed. If any locus yielded no reliable genotype, that locus was treated in the analysis as missing data.

### Sexing

The sex of individuals identified through microsatellite genotyping was determined genetically by amplifying sex specific fragments from DBY intron 7 and DBX intron 6. Primers used for amplification are canid specific and amplify two differently sized fragments in males and one in females [40]. PCR amplification consisted of one multiplexed 10 µl reaction with primer concentrations of 0.1–0.2 uM, 3 µl template DNA and 1 × Qiagen multiplex PCR master mix (QMP) primer (Additional file 1: Table S1). The PCR profile had an initial denaturation at 95 °C for 15 min followed by a touchdown profile with 20 cycles of denaturation at 94 °C for 30 s, annealing at 60 °C for 90 s with a 0.2 °C decrease in temperature after each cycle and extension at 72 °C for 60 s, followed by 20 more cycles with annealing at 55 °C and a final extension step at 60 °C for 30 min. Amplified fragments were visualized on a 2% agarose gel.

### Individual identification

Individual identification analysis was done with the software *Gimlet v.1.3.3* and *Cervus* 3.0.7 [41]. Matching multilocus genotypes were assumed to represent the same individual. Due to the likelihood of genotyping errors due to the low quality and quantity of the non-invasively collected DNA, we allowed maximum two mismatching alleles (one at each of two loci) between genotypes for them to be considered to have originated from the same individual. All assembled consensus individual genotypes were successfully genotyped at a minimum of 11 loci (median 17 loci).

Molecular checks for potential dog samples were necessary to ascertain that the collected fecal samples were indeed from wolves as the droppings and tracks of wolves and domestic dogs can look very similar in the field. Dog fecal samples were identified against the dog reference samples using either factorial correspondence analysis (FCA) in the software *Genetix* [42] or *NewHybrids* [43] and excluded from further analysis.

### Pedigree fitting

The pedigree was estimated using the MasterBayes package in R, which implements a Monte Carlo Markov chain (MCMC) sampling approach to simultaneously estimate the pedigree alongside other population-level parameters in a Bayesian framework [23]. Opportunistically or non-invasively collected genetic data can be challenging for constructing pedigrees [23–25]. Several precise pedigree reconstruction techniques are able to overcome these data limitations by making assumptions on unknown variables, but this can be problematic as it risks propagating errors into the parentage assignments [24, 26]. Moreover, resolving the direction of parent–offspring relationships in a multigenerational pedigree is an unsolved challenge in for exact pedigree reconstruction methods [25]. The Bayesian approach provides a robust solution to improve reliability of parentage assignment in incompletely sampled populations using low-quality DNA sources [23–25]. Unknown parameters such as the unsampled population size and genotyping error rate are modelled including uncertainty, avoiding assumptions and thus reducing bias in parentage assignments, and the iterative sampling of pedigree configurations enables identification of the most likely directions of relationships between individuals without demographic information [23–25].

Allele frequencies were taken from a previously published data-set covering the entire range of the Finnish wolf population [28] (Additional file 1, n = 185 individuals) to avoid skewing the likelihoods of allele sharing by descent in the small, potentially closely related population of the current study. Alleles in the SW Finland sub-population that were not present in the Finland-wide sample were added manually at low frequency. All pedigrees treated genotyping error rate as two distinct components, drop-out rate (non-amplification, E1) and stochastic error (genotype wrongly scored, E2) [44]. All pedigrees used the same priors on unsampled population size, broad lognormal distributions with means in the likely real parameter range (for males µ = log(7), σ = 0.5; for females, µ = log(2), σ = 0.5), and all MCMC chains were thinned every 2 iterations. Individuals that mismatched offspring at more than 4 loci were excluded as potential parents; the main pedigree was robust to a range of mismatch thresholds. For each pedigree, the MCMC sampling was tuned to achieve the recommended acceptance rate for efficient mixing (30–50%). Individuals known to have died at younger than 2 years of age (the earliest known reproduction of wild wolves [45]) were excluded as potential parents and the pedigree was checked for legality (no loops) at each iteration. The data was ordered to make later-observed individuals more likely to be fitted as offspring, but no explicit temporal information was available to inform age or cohort structure. To optimize our use of the available data and improve the mixing of the Markov chains, the final pedigree was obtained in several stages: (1) deriving a preliminary pedigree to inform estimation of genotyping errors; (2) estimating error rates; (3) restricting candidate parents to likely individuals; (4) fitting pedigrees using these estimated parameters; (5) averaging out stochasticity in the sampling to obtain a robust main pedigree; (6) assessing the biological sensibility of all assignments.

First, a preliminary pedigree (step 1) was estimated using the consensus individual genotypes with fixed error rates (E1 = 4%, [46]; E2 = 2.2%, [47]), run for 20,000 iterations with a burn-in of 2000, and the modal pedigree extracted from the sampled posterior distribution of pedigrees. Parentages with over 90% confidence from this preliminary pedigree were used as the input pedigree for the error rate estimation (step 2) from the per-sample dataset, which thus used information both from repeated samples of individuals and family relationships. Error rates were estimated for each locus. The pedigree was kept fixed, priors on the error rates were broad with means as above (shape parameters of beta distribution: E1, α = 4, β = 100; E2, α = 2, β = 100) and the model was initiated at a high but realistic value for both error types (0.1) to fully explore parameter space. In pedigree estimation (steps 3 and 4), the estimated error rates were used for individuals represented only by scat samples, which made up the majority of both repeat samples and samples from individuals with preliminary pedigree information. For tissue samples, error rates could not be reliably estimated from this data (only 6 individuals had more than one tissue sample), so a constant E1 and E2 from previous work for a range of tissue types were used across the panel [47]. To further reduce the dimensionality of the main pedigree estimation problem and hence increase confidence in distinguishing between parents with numerically similar likelihoods, before fitting the final pedigree, we first identified potential breeding individuals (step 3). This model used fixed, derived error rates from step 2 and was run 20 times, each run using 25,000 iterations after a short burn-in of 1000 to ensure sufficient exploration of probability space. From across all 20 modal pedigrees, all individuals assigned in ≥ 5% of parentages were taken as “possible breeders” in the main pedigree estimation.

For the main pedigree fit (step 4), we restricted potential parents to be only the identified possible breeders and used a chain of 30,000 iterations with a burn-in of 3000. To make the ultimate output pedigree robust to the stochasticity inherent in a random sampling scheme, this main model was run 20 times and the modal pedigree extracted from each run including likelihoods for each assignment. For each offspring, the average likelihood of each parent pair was calculated across these 20 pedigrees (step 5) and the most likely overall pair assigned as parents in the main pedigree. These assignments were robust to lability among poorly resolved relationships. However, due to a lack of accurate temporal information and low diversity at many of the microsatellite loci, some assignments required manual correction post hoc (step 6) according to biological understanding (“biologist corrections” [48]). These corrections were based on likely birth years inferred from the obtained pedigree, a minimum age of 2 years at reproduction, and wolves’ mating system of only one pair in each territory producing a litter of several cubs [45]. Because reliable age data was only available for very few individuals and could thus not be used to constrain candidate parents, some assignments were found to be temporally impossible (insufficient time for given number of generations) based on birth years (cohort) inferred from sample dates for individuals throughout the pedigree structure. Such anachronisms were manually removed (mothers of 2 offspring, details in Additional file 1). In addition, certain medium-confidence (70–80%) parent pairs were biologically unlikely, appearing in the pedigree as parents to only one individual, whereas all reliably assigned pairs (with at least one offspring assigned to both parents with over 95% likelihood in the final pedigree) were represented by several offspring. In all such cases of uncertain assignments, parentages could be confirmed or offspring could manually be reassigned to a reliably identified breeding pair, in the same territory as the offspring, whose genotypes were consistent with the offspring’s.

### Simulation tests of assignment accuracy

To explore constraints on the reliability of assignments, pedigrees were fitted to simulated genotypes using different numbers of microsatellite markers. Genotypes were simulated using the *simgenotypes* function in *MasterBayes* for the sampled population, using the configuration of the final pedigree as the “true” pedigree and using the same base allele frequencies, error rates and set of possible parents as above. Genotypes were simulated for panels of 5, 10, 17, 20, 25, 30 and 34 loci, randomly sampled without replacement (within each group of up to 17 loci) from the real markers [following 26]. For each panel size, 10 sets of genotypes were simulated, and for each simulated genotype set, a pedigree was fitted according to steps 3–5 above. This output pedigree was compared to the true (input) pedigree.

### Analysis and pedigree interpretation

All analysis was carried out in R [49]. Expected heterozygosity (H_E_) was calculated using the library *pegas* [50], population-level inbreeding based on allele frequencies using the library *adegenet* [51], and inbreeding based on pedigree links using the library *pedigree* [52]. The simulated pedigrees were assessed in terms of the number of confident assignments made, and the number of confident assignments that were correct, i.e. matched the input pedigree. Unless otherwise stated, we term assignments “confident” at over 80% likelihood. All descriptive statistics are given as mean ± standard error.

From the pedigree, we interpreted further details of breeding timing and dispersal routes using knowledge of the biology of the system and the place and time of collection of each sample. Likely breeding years, and hence birth years of new cubs, were inferred from the simultaneous appearance of sibling groups, supported by the estimated ages of killed young wolves (where available). Individuals assigned to two unsampled parents were assumed to be immigrants. All these interpretations are henceforth presented as “likely” scenarios, as we cannot rule out that individuals were present at other times or places but simply avoided detection, which could affect interpretation.

## Results

In total, 516 scat samples were collected in the study area by at least 48 named collectors. Of these samples, 10 were identified as non-wolf (dog or other canid). Of the wolf samples, 368 were successfully genotyped at ≥ 10 loci and could be assigned to a known or new individual. Among all samples, including those from other sources, we identified 81 distinct individuals, represented by 1–19 samples each (median 4 samples per individual). Of these, 23% of samples required a second (78 samples) or third (6 samples) triplicate PCR run to obtain a sufficiently complete genotype.

Of the 81 individuals, 46 (57%) were confidently (≥ 80% likelihood) assigned both a known mother and known father, while only 11 (14%) could not be confidently be assigned any parent (Table 2). Of all 162 possible assignments (mother and father of 81 individuals), 116 (72%) were made with over 90% likelihood and only 7 (4%) with less than 50% likelihood. Of the confident assignments, 5 were to unsampled parents (3 sires and 4 dams, including 2 unsampled pairs). After making manual adjustments based on biological understanding for one or both parents of 13 offspring and confirmations for a further 13 (details in Additional file 1), the final pedigree gave credible full parentages for 70 of the 81 individuals (86%) and left only 2 individuals (2%) with no credible assignment for either parent (Table 2). Manual changes were particularly necessary among a group of historical individuals (AUL-002 to AUL-009; 7 of these 8 individuals had assignments altered or confirmed) not recorded since 2009 and with no confident links to other sampled individuals. Relationships within this historical group were poorly resolved and they are therefore not included in our analysis of recent pack configurations.

**Table 2:** Summary of the number of confident assignments made in the pedigree fit (main pedigree) and following knowledge-based confirmations of low-confidence assignments or adjustments of temporally impossible or biologically unlikely assignments (final pedigree). Complete individual assignments from the main and final pedigrees are given in Additional file 1

	Nr. of individuals	
	Main pedigree	Final pedigree
Both parents	50	70
Only mother	6	4
Only father	14	5
Neither	11	2

Panel-wide H_E_ was 0.58 ± 0.04 (range per locus 0.20–0.78). Although inbreeding appeared high at the population level on the basis of allele frequencies (across all individuals, mean F = 0.15 ± 0.01, range 0.05–0.47), the pedigree structure gave evidence of only one recent inbreeding event between a grandfather and granddaughter (which gives a pedigree-based F = 0.007).

**Fig. 2:**
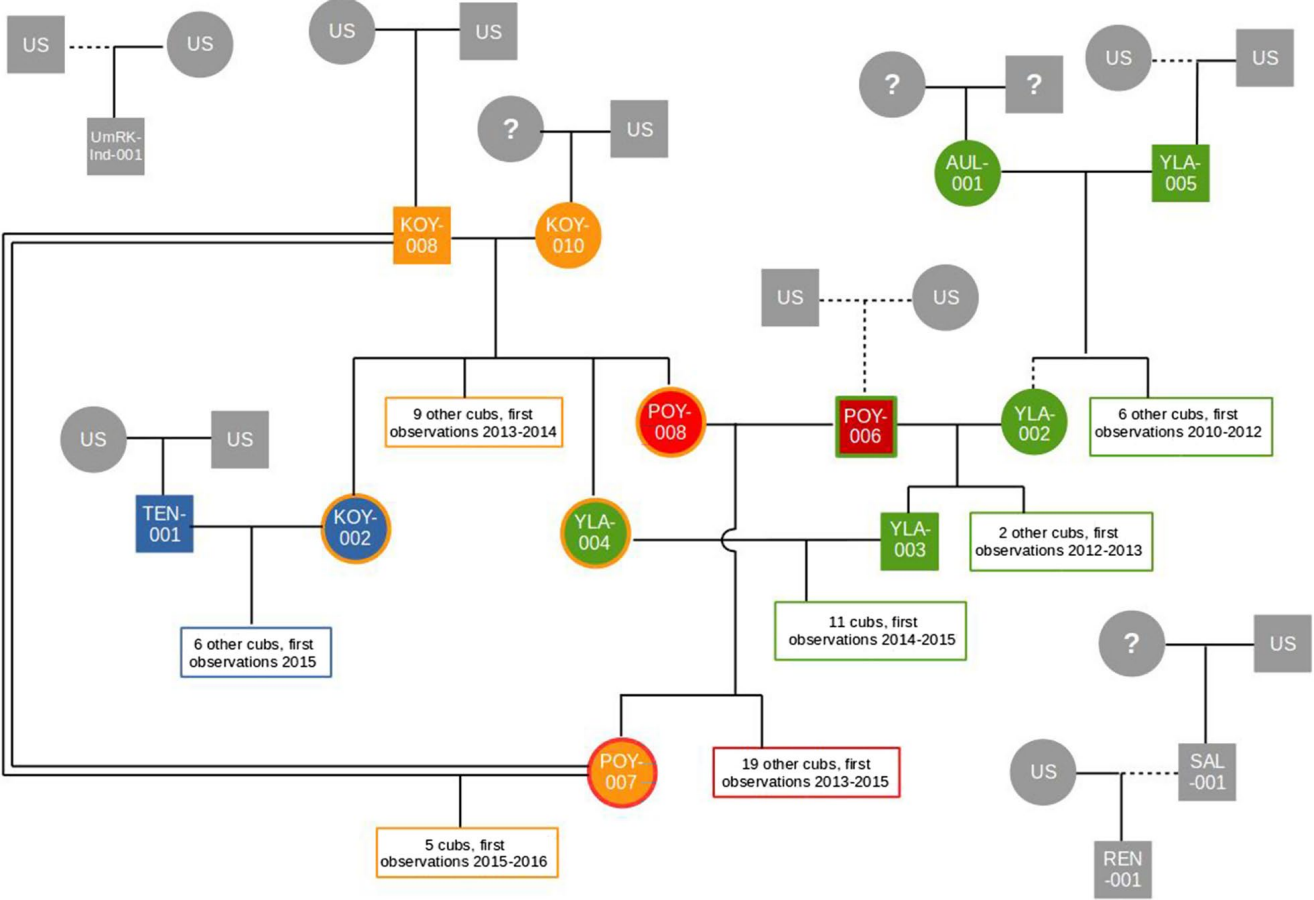
The final pedigree configuration, with known breeders shown individually and other individuals shown in sibling groups. Squares denote males and circles females. Symbol colours denote the individuals’ breeding pack and the symbol outline colours indicate their natal pack. Solid lines show links with over 95% confidence and dotted lines 80–95% confidence. The double line indicates inbreeding. Assignments to some cubs in the sibling groups were manually altered; full details of the pedigree are given in Additional file 1

**Fig. 3:**
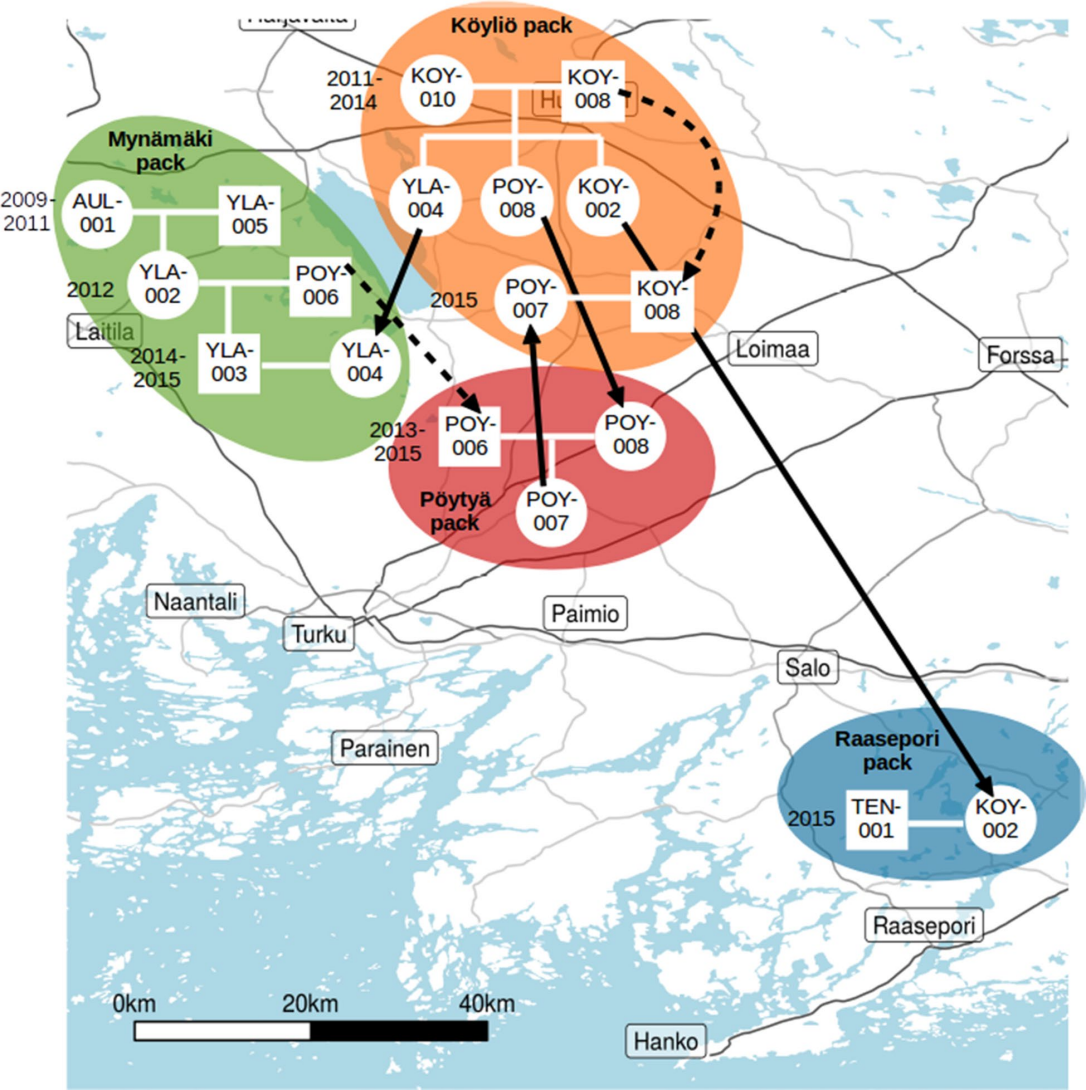
Schematic representation of pack dynamics, as indicated by changes in the breeding pairs, in the study area over the study period. Only known breeders are shown; circles denote females and squares males. Inferred years of reproduction are shown next to each alpha pair. Solid arrows show individuals’ dispersal from natal locations and dotted arrows show mate switching and dispersal of established breeders. Individuals with no parents indicated have uncertain or unsampled parents; see Fig. 2 for the finalized pedigree and Additional file 1: Table S2 for full details of the pedigree fit. Pack areas are approximate and do not reflect real territory boundaries; they are only drawn for illustration. True locations of all samples are shown in Fig. 1

Using knowledge about the biology of the system combined with sample-level location and temporal data, we were able to reconstruct temporally explicit pack dynamics and individual movements over the study period (Fig. 3). Throughout the study period, breeding pairs and timing of reproductive events identified from the pedigree were consistent with available field observations of individuals and packs. Litters were produced by 7 breeding pairs across 4 pack territories, including the split of one pack territory into two and one pair that established a new territory ~ 150 km south-east of the core monitoring area during the course of the study (Figs. 2, 3). The cumulative number of identified offspring produced by each pair ranged from 3 to 18, with all sibling groups likely spread over 1–3 years of breeding pair tenure. Dispersal was common, with gene flow observed to and/or from all packs.

Adults showed a range of strategies to secure breeding opportunities, with some indications of sex differences. Among adults whose natal packs were known, one female and one male inherited breeding positions, whereas four females but no males dispersed within the area before establishing as breeders. Among individuals of unknown origin, six were males and two females, of which all but two males established as breeders. That these individuals’ parents were not represented in our samples may be because they are long-distance immigrants, with parents outside the sampling area, or because they represent the earliest samples collected in our study (particularly for the earliest breeders), with parents outside the sampling period. The longest-established pack (Mynämäki territory, Figs. 2, 3) has had three distinct identified breeding pairs since 2009, with the latter two consisting of an offspring of that pack, i.e. inheriting the breeding position, with a dispersing mate. In the first such case, a daughter reproduced once with a likely immigrant male in 2012 (after the initial breeding female was shot the previous winter). In the second case, 2 years later, a son reproduced with a female originating from the neighbouring Köyliö pack) (Fig. 2). The likely immigrant male from 2012 subsequently reproduced with another female for 3 years in a neighbouring area, establishing the Pöytyä territory. This indicates that in 2012 the territorial dynamics were still rather unestablished in the area.

**Fig. 4:**
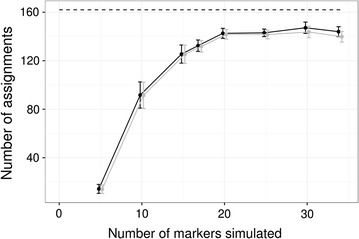
Assignment success for pedigrees fitted to simulated genotypes using a range of marker panel sizes. For each panel size, n = 10 simulated genotype sets. The total number of confident assignments are shown in black, and the number of confident assignments that were correct (captured the input pedigree used to simulate the genotypes) are shown in grey. Points and error bars show the mean ± 1 se. The black dotted line indicates the maximum number of assignments (162: mother and father of 81 individuals)

In contrast to Mynämäki, offspring in the Köyliö pack have been produced by only one male, a likely immigrant, paired with two different females (a likely immigrant for 2011–14, followed in 2015 by the male’s granddaughter dispersing from the Mynämäki pack). Three daughters of the first Köyliö pair dispersed over several years to breed in neighbouring territories with inheriting (Mynämäki pack), likely immigrant (Raasepori pack) or adult disperser (Pöytyä pack) males. Thus, in 2014, the three reproductive females in the core area were a mother and two of her daughters (Fig. 2).

The pedigree revealed two occasions of mate switches; in both cases a male’s initial female disappeared from the record but we do not have direct evidence of mortality. It is therefore unclear whether these switches were active mate choice by the male or a response to losing an established partner. There were no instances of new breeding pairs being established between existing pack members. Full details of the interpretation of pack dynamics are given in Additional file 1.

To examine the statistical robustness of our parentage assignments, we repeated the pedigree fitting on a population of simulated genotypes based on the data. This test showed that 17 markers, i.e. the size of the current panel, gave a mean of 131.9 correct and confident assignments (81.4% of 162 possible assignments). Increasing the panel size to 20 slightly improved accuracy to a mean of 141.7 (87.5%) correct, confident assignment, but further increases up to 34 loci made no significant improvement (linear models: change to number of correct assignments with 20 compared to 17 markers, 3.27 ± 1.05, t = 3.125, p = 0.006; effect of number of markers from 20 to 34 on number of correct assignments − 0.06 ± 0.20, t = − 0.32, p = 0.753) (Fig. 4). Hence, while our pedigree-fitting pipeline is not able to completely resolve all pedigree uncertainties, the confidently-resolved relationships in the empirical data are most likely reliable: at all simulated panel sizes, very few confident assignments were made that were not correct (Fig. 4). At the current panel size of 17 loci, a mean of 132.4 parents were confidently assigned, of which 131.9 were correct, giving a false positive rate of only 0.3% among confident assignments in the simulation.

## Discussion

Here, we have described the pack dynamics of a newly-established and expanding wolf population in south-west Finland based on a collaborative genetic sampling framework in which stakeholders representing a range of interests contributed to knowledge generation. We subsequently used this non-invasively collected genetic data for multi-generational pedigree reconstruction, allowing us to characterize reproductive dynamics and individual dispersal patterns in and between the local wolf packs. This ongoing study provides an excellent opportunity to closely follow current changes in pack structure and reproductive decisions in a dynamic population. The reconstructed pedigree shows a closely interrelated population consisting of sibling groups from 7 breeding (alpha) pairs across four established packs in 2009–2015, with individuals dispersing/moving between all the packs. Despite this, there was little direct inbreeding among the range of strategies utilized to obtain and maintain reproductive opportunities. These results illustrate the value of molecular data for elucidating reproductive dynamics and movements of packs and individuals in detail that not achievable through on-the-ground monitoring alone. Our analytical routine maximized confidence in the final pedigree despite the potentially low quality of the primarily faecal DNA used, the collection of field data mainly by volunteers, and the inherent challenges of multigenerational pedigree reconstruction in incompletely sampled populations [25]. While non-invasive genetic monitoring of individuals is a well-established management tool [15, 20, 37, 53, 54], our extension of previous approaches allows us to detail ongoing changes in the size and structure of packs and the population, providing accessible, unbiased information that is much-needed in the fierce debate concerning management of the grey wolf.

### Characterizing pack dynamics

We found that all packs in this small recovering population are related through several different pathways. While calculations based on allele frequencies suggested substantial inbreeding, the pedigree structure indicated that the high interrelatedness is largely due to the population being mainly composed of related sibling groups, rather than a result of recent inbreeding events. Despite the newly-established population expanding to four simultaneously reproducing pairs in recent years, the first instance of direct inbreeding was only detected in 2015. Here, the female was the male’s granddaughter, a level of relatedness (r = 0.25) at which wolves have previously been shown to avoid inbreeding [between half-sibs; 55]. The rarity of inbreeding during the study period suggests that inbreeding avoidance occurs in this population, particularly as most potential mates are related and many detected immigrants have become breeders; inbreeding avoidance occurs in other grey wolf populations as well as in other canids [21, 55, 56]. However, it is important to note that we have insufficient information to rule out the possibility that the first breeding individuals in the data (in the first pairs in Mynämäki and Köyliö) could have been relatives. Relatedness among the founders would for example contribute to the high apparent inbreeding suggested by allele frequencies. In addition, in this small population in which only a small proportion of individuals are likely to breed, genetic drift is expected to decrease diversity over time, and opportunities for inbreeding may become more frequent as the population expands [28]. Despite these influences tending to reduce diversity, our results suggest that natural mechanisms such as inbreeding avoidance can at the same time contribute to limiting the potential negative effects of associated inbreeding depression [as in 56], supporting the short-term viability of the SW Finland wolf population. Continued monitoring of this expanding population affords a rare opportunity to track ongoing changes in inbreeding dynamics, which could enable the design of management actions to provide opportunities for outbreeding, such as dispersal corridors, and hence maintain diversity and support reproduction [56, 57].

Potential and established breeders used a broad range of strategies to obtain and maintain reproductive opportunities, with inbreeding apparently the least preferred option after dispersal to existing or new packs, inheriting breeding positions in the natal pack, or (for established breeding males) mate switching. The only instance of inbreeding occurred when a dispersing female joined an established male, whose previous mate disappeared from our records the year before the new litter was recorded. This diversity of breeding strategies and strong inbreeding avoidance mirrors findings from a reintroduced North American wolf population [56]. Overall, females dispersed between known packs more frequently than males, although our pedigree shows that this may not be sufficient to ensure inbreeding avoidance in a densely occupied habitat. In contrast, males that became established as breeders during the study tended to come from unknown natal packs, i.e. likely long-distance dispersers. This pattern of shorter female and longer male dispersal is also seen in other canids [African wild dogs *Lycaon pictus*, 58]. Together with the observation that there were more novel breeding opportunities for females in our population, this suggests that while dispersal at the local scale may be sufficient to secure a breeding position for females, males must disperse over longer distances. Such immigration is extremely valuable in maintaining genetic diversity and population viability, particularly in small and interrelated populations [56, 57]. Dispersal is common in other parts of the expanding Finnish wolf population: in a 6-year study, over half of tracked non-alpha individuals dispersed from their natal pack, but with only a minority of dispersers (33%) reproducing in a new territory [29]. Moreover, our female-biased local dispersal but male-biased immigration suggests that harvesting breeding males may limit population expansion more than removing females, which could be more easily replaced locally. In any case, continued population expansion does appear to be possible: two new packs were established (Pöytyä and Raasepori) during the study, and suitable breeding territories may yet remain unfilled. Such insights into current reproductive dynamics illustrates the value of applying genetic monitoring approaches to a newly-establishing population, in contrast to previous work focusing on established populations and historical patterns.

These insights into the behaviour of SW Finland wolves are underpinned by the robustness of our pedigree: 74% of all parentage assignments in the final pedigree were made with high confidence, even in this interrelated population with relatively few founders and consequently limited genetic diversity (H_E_ of 0.58, compared to 3-year temporal estimates across the Finnish population during 1995–2009, H_E_ > 0.67, [28]). Of the low-confidence assignments, 15 could be confirmed based on biological understanding of the system, but conversely, such interpretation suggested that 4 confident assignments and 16 low-confidence assignments were biologically unlikely. The majority of these manual changes were made to parents that were most likely too young or too many generations back, once likely birth years had been interpreted from the pedigree configuration of sibling groups and their detection dates. Reliable age data would contribute substantially to increasing confidence in our pedigree structure, but this is a challenge to obtain from non-invasive samples. Due to the inherent opportunism in production and detection of faecal material, an individual may be in the population long before or after its first or last detection, even when the sample collection protocol is systematic. Therefore, we did not have sufficiently accurate chronological data for most individuals (e.g. cohort or death date) with which to explicitly constrain potential parent–offspring relationships. It would be valuable to investigate whether faecal lobe size could be used to indicate the age of individual wolves, as has been done in non-invasive genetic studies of western gorillas [59].

Despite these practical challenges, a non-invasive sampling scheme involving citizen scientists provides a productive tool to underpin management decisions for contentious populations such as the SW Finland wolves. Enabling local people from different backgrounds to contribute concretely to data collection can mitigate the perceived conflict over who holds ownership of information [8, 11, 13]. Moreover, direct involvement with monitoring can increase the public’s understanding of wolf biology, which contributes to mitigating conflicts with the predator [7, 8].

### Pedigree reconstruction from low-quality genetic material

In our study, the drawbacks of a non-invasive sampling scheme were dealt with effectively by combining it with Bayesian estimation of the pedigree. The lack of temporal resolution, individuals missing from the samples, and potential for high error rates associated with non-invasive sampling of genetic material [36] are also inherent constraints on exact pedigree reconstruction techniques [23–25, 27]. The MasterBayes estimation framework currently provides the most utilitarian solution to this problem, with the possibility of using chronological or other phenotypic data (where available), to improve relationship resolution while retaining the ability to assign unsampled parents [27] and thus being robust to incomplete sampling of the population. However, even in this flexible pedigree-fitting routine, it remains a challenge to separate between parent–offspring and sibling relationships (both r = 0.5) where one parent is unsampled and there is no temporal information to inform the direction of the relationship [23, 25]. For example, if the true father is missing from the data, the brother of an individual is likely to be assigned as that individual’s father [23]. This may have contributed to the difficulty in confidently resolving relationships among the historical members of the Mynämäki pack. The earliest litters of individual AUL-001, a long-standing breeding female in that pack, were produced before our systematic monitoring began (see SI), likely with an unsampled male, reducing our confidence in distinguishing sibships from parentages. Similarly, individuals SAL-001 and REN-001 were assigned as father and son with an unsampled mother, but may in fact be brothers with both parents unsampled. This uncertainty must be borne in mind when interpreting this type of genetic monitoring data from incompletely sampled populations, as in the majority of conservation applications.

Nonetheless, our simulations showed that at present, our panel of 17 established microsatellites [28, 33, 34] is sufficient to make over 80% of assignments confidently and correctly, and that this could be increased to a maximum of almost 90% by adding 3 extra loci. Broadly, the main limitation on inference from the pedigree was confidence rather than accuracy of assignments: 10–20% of the simulation assignments were made with low confidence, whereas for panels larger than 5 loci only an average of 2.4% of assignments were incorrect. Confidence is likely limited in part by population structure and in part by assumptions in the analysis. Firstly, the close relatedness structure and limited genetic diversity of this population make it difficult to choose between genetically similar potential parents [60], often with descendants or siblings identified as parents. For example, AUL-001 carried common alleles at many loci, giving several other individuals, particularly her offspring, similar likelihoods of having produced her offspring. Secondly, we estimated genotyping error rates conservatively—per sample, not accounting for consensus genotypes based on multiple samples—and thus they are likely too high. This relaxation of the assumption that genotypes are true reduces the chance of erroneous assignments, but also reduces confidence as mismatches could be due to error either in the genotype or the assignment. Using a different marker type, single-nucleotide polymorphisms (SNPs), has the potential to improve confidences by reducing genotyping error due to the shorter sequences amplified [61, 62]. SNP-based pedigree reconstruction has already generated management-relevant information on reproductive behaviour in brown bears (*Ursus arctos*) [22] and a 96-locus multiplex SNP panel suitable for non-invasively collected genetic material has recently been developed for individual identification of wolves [60]. However, the limited variation per SNP locus compared to multiallelic microsatellites, possibly exacerbated by relatively high rates of missing data (up to 10% of samples per locus; [61]), means that pedigree reconstruction requires a large number of markers to capture sufficient variation to resolve relationships. This sort of study would likely require a larger number of markers than currently established for wild wolves: using non-invasive samples from a wide-ranging brown bear population, 96 SNPs were sufficient to identify 433 individuals but to assign at least one confident parent to only 82 of these (19%; [22]).

## Conclusions

The pipeline we have developed provides a reliable framework for pedigree reconstruction in this newly-established, incompletely and non-invasively sampled, multigenerational population. While some uncertainty remained in the computational outcome, this could mostly be satisfactorily resolved through manual corrections based on knowledge gained from the output pedigree. From the final pedigree, many aspects of population change of direct relevance to management decisions can be interpreted, such as dispersal and reproductive strategies, reconfigurations of mating pairs following mortality and average breeding tenure. For example, understanding dispersal patterns allows inference of likely responses of packs to the removal of breeding individuals, and hence the potential to manage gene flow between packs to some extent by targeting certain individuals during population-control hunting. Ongoing data collection will provide an opportunity to test such measures by examining changes in pack structure as a consequence of several breeding individuals being legally shot in 2016. These successes and opportunities support the continued use and planned expansion of this monitoring technique in this wolf population. If sampling and analysis can be conducted frequently enough, pack dynamics could be tracked in close to real time, perhaps even enabling prediction of certain reproductive events. Understanding how pack structure changes in the early stages of recolonisation illustrates how natural establishments and management interventions might proceed in other conservation contexts, such as reintroductions [16, 56]. As well as providing management-relevant information, the pedigree approach to characterizing population dynamics provides a qualitative description of the history and current structure of the population that may be more likely to be understood and accepted as unbiased by different stakeholders in the conflict. Unbiased information and public involvement are crucial to help move the fierce debate away from generalizations and emotion-driven claims towards a more evidence-based discussion, facilitating effective decision-making around the management of the wolves in this area and other re-establishing carnivores in densely-populated areas globally.

## Additional file

**Additional file 1.** Additional details on methods and results are given in this supplementary file: a complete description of the molecular methods (**Appendix 1**); a full interpretation of pack dynamics (**Appendix 2**); details on microsatellite and primer sequences (**Table S1**); and the full pedigree outputted from the analysis pipeline and manual adjustments made to it (**Table S2**).

## References

[1] Estes JA, Terborgh J, Brashares JS, Power ME, Berger J, Bond WJ (2011). Trophic downgrading of planet Earth. Science.

[2] Ripple WJ, Estes JA, Beschta RL, Wilmers CC, Ritchie EG, Hebblewhite M (2014). Status and ecological effects of the world’s largest carnivores. Science.

[3] Loe J, Roskaft E (2004). Large carnivores and human safety: a review. Ambio.

[4] Penteriani V, Delgado MM, Pinchera F, Naves J, Fernandez-Gil A, Kojola I (2016). Human behaviour can trigger large carnivore attacks in developed countries. Sci Rep..

[5] Chapron G, Kaczensky P, Linnell JDC, von Arx M, Huber D, Andrén H (2014). Recovery of large carnivores in Europe’s modern human-dominated landscapes. Science.

[6] Smith DW, Peterson RO, Houston DB (2003). Yellowstone after wolves. Bioscience.

[7] Glikman JA, Vaske JJ, Bath AJ, Ciucci P, Boitani L (2011). Residents’ support for wolf and bear conservation: the moderating influence of knowledge. Eur J Wildl Res.

[8] Hiedanpää J (2013). Institutional misfits: law and habits in Finnish wolf policy. Ecol Soc..

[9] Boitani L, Mech LD, Boitani L (2003). Wolf conservation and recovery. Wolves behavior, ecology, and conservation.

[10] Kojola I, Helle P, Heikkinen S, Lindén H, Paasivaara A, Wikman M (2014). Tracks in snow and population size estimation: the wolf *Canis lupus* in Finland. Wildl Biol..

[11] Hiedanpää J, Salo M, Kotilainen J (2015). Teleodynamics and institutional change: the hardship of protecting the Amur tiger, big-leaf mahogany, and gray wolf. J Nat Cons.

[12] Linnell JDC, Andersen R, Andersone Z, Balciauskas L, Blanco JC, Boitani L (2002). The fear of wolves: a review of wolf attacks on humans. NINA Oppdragsmeld.

[13] Pohja-Mykrä M, Kurki S (2014). Strong community support for illegal killing challenges wolf management. Eur J Wildl Res.

[14] Kojola I, Hallikainen V, Mikkola K, Gurarie E, Heikkinen S, Kaartinen S, Nikula A, Nivala V (2016). Wolf visitations close to human residences in Finland: the role of age, residence density, and time of day. Biol Cons.

[15] Waits LP, Paetkau D (2005). Noninvasive genetic sampling tools for wildlife biologists: a review of applications and recommendations for accurate data collection. J Wildl Man.

[16] Bergl RA, Vigilant L (2007). Genetic analysis reveals population structure and recent migration within the highly fragmented range of the Cross River gorilla (*Gorilla gorilla diehli*). Mol Ecol.

[17] Schwartz MK, Monfort SL, Long RA, MacKay P, Zielinski WJ, Ray JC (2008). Genetic and endocrine tools for carnivore surveys. Non-invasive survey methods for carnivores.

[18] Smith O, Wang J (2014). When can noninvasive samples provide sufficient information in conservation genetics studies?. Mol Ecol Res.

[19] Lucchini V, Fabbri E, Marucco F, Ricci S, Boitani L, Randi E (2002). Noninvasive molecular tracking of colonizing wolf (*Canis lupus*) packs in the western Italian Alps. Mol Ecol.

[20] Stenglein JL, Waits LP, Ausband DE, Zager P, Mack CM (2011). Estimating gray wolf pack size and family relationships using noninvasive genetic sampling at rendezvous sites. J Mammal.

[21] Caniglia R, Fabbri E, Galaverni M, Milanesi P, Randi E (2014). Noninvasive sampling and genetic variability, pack structure, and dynamics in an expanding wolf population. J Mammal.

[22] Norman AJ, Spong G (2015). Single nucleotide polymorphism-based dispersal estimates using noninvasive sampling. Ecol Evol..

[23] Hadfield JD, Richardson DS, Burke T (2006). Towards unbiased parentage assignment: combining genetic, behavioural and spatial data in a Bayesian framework. Mol Ecol.

[24] Koch M, Hadfield JD, Sefc KM, Sturmbauer C (2008). Pedigree reconstruction in wild cichlid fish populations. Mol Ecol.

[25] Jones A, Small C, Paczolt K, Ratterman N (2010). A practical guide to methods of parentage analysis. Mol Ecol Res..

[26] Aykanat T, Johnston SE, Cotter D, Cross TF, Poole R, Prodőhl PA, Reed T, Rogan G, McGinnity P, Primmer CR (2014). Molecular pedigree reconstruction and estimation of evolutionary parameters in a wild Atlantic salmon river system with incomplete sampling: a power analysis. BMC Evol Biol.

[27] Walling CA, Pemberton JM, Hadfield JD, Kruuk LEB (2010). Comparing parentage inference software: reanalysis of a red deer pedigree. Mol Ecol.

[28] Jansson E, Ruokonen M, Kojola I, Aspi J (2012). Rise and fall of a wolf population: genetic diversity and structure during recovery, rapid expansion and drastic decline. Mol Ecol.

[29] Kojola I, Aspi J, Hakala A, Heikkinen S, Ilmoni C, Ronkainen S (2006). Dispersal in an expanding wolf population in Finland. J Mammal.

[30] Kojola I, Kaartinen S, Hakala A, Heikkinen S, Voipio H-M (2009). Dispersal behavior and the connectivity between wolf populations in Northern Europe. J Wildl Man..

[31] Barja I, de Miguel FJ, Barcena F (2004). The importance of crossroads in faecal marking behaviour of the wolves (*Canis lupus*). Naturwissenschaften.

[32] Gurarie E, Suutarinen J, Kojola I, Ovaskainen O (2011). Summer movements, predation and habitat use of wolves in human modified boreal forests. Oecologia.

[33] Fredholm M, Wintero AK (1995). Variation of short tandem repeats within and between species belonging to the canidae family. Mamm Gen..

[34] Francisco LV, Langston AA, Mellersh CS, Neal CL, Ostrander EA (1996). A class of highly polymorphic tetranucleotide repeats for canine genetic mapping. Mamm Gen..

[35] Morin PA, Chambers KE, Boesch C, Vigilant L (2001). Quantitative polymerase chain reaction analysis of DNA from noninvasive samples for accurate microsatellite genotyping of wild chimpanzees (*Pan troglodytes verus*). Mol Ecol.

[36] Taberlet P, Luikart G, Waits LP (1999). Noninvasive genetic sampling: look before you leap. Trends Ecol Evol.

[37] Arandjelovic M, Head J, Kuhl H, Boesch C, Robbins MM, Maisels F, Vigilant L (2010). Effective non-invasive genetic monitoring of multiple wild western gorilla groups. Biol Cons.

[38] Jansson E, Harmoinen J, Ruokonen M, Aspi J (2014). Living on the edge: reconstructing the genetic history of the Finnish wolf population. BMC Evol Biol.

[39] Stansbury CR, Ausband DE, Zager P (2014). A long-term population monitoring approach for a wide-ranging carnivore: noninvasive genetic sampling of gray wolf rendezvous sites in Idaho, USA. J Wildl Man..

[40] Seddon JM (2005). Canid-specific primers for molecular sexing using tissue or non-invasive samples. Cons Gen..

[41] Kalinowski ST, Taper ML, Marshall TC (2007). Revising how the computer program CERVUS accommodates genotyping error increases success in paternity assignment. Mol Ecol.

[42] Belkhir K, Borsa P, Chikhi L, Raufaste N, Bonhomme F. GENETIX 4.05, logiciel sous Windows TM pour la génétique des populations. Laboratoire Génome, Université de Montpellier II, Montpellier (France). 1996–2004.

[43] Anderson EC, Thompson EA (2002). A model-based method for identifying species hybrids using multilocus genetic data. Genetics.

[44] Wang J (2004). Sibship reconstruction from genetic data with typing errors. Genetics.

[45] Mech LD, Boitani L (2003). Wolves: behavior, ecology, and conservation.

[46] Kuismin J. Assessing wolf’s (*Canis lupus*) population size in south-western Finland using genetic methods. Master’s thesis, University of Turku, Finland; 2015.

[47] Scandura M, Capitani C, Iacolina L, Marco A (2006). An empirical approach for reliable microsatellite genotyping of wolf DNA from multiple noninvasive sources. Cons Gen..

[48] Knapp SM, Craig BA, Waits LP (2009). Incorporating genotyping error into non-invasive DNA-based mark—recapture population estimates. J Wildl Man..

[49] R Core Team. R: A language and environment for statistical computing. R Foundation for Statistical Computing, Vienna; 2013.

[50] Paradis E (2010). Pegas: an R package for population genetics with an integrated—modular approach. Bioinformatics.

[51] Jombart T (2008). Adegenet: a R package for the multivariate analysis of genetic markers. Bioinformatics.

[52] Coster A. Pedigree: pedigree functions. R package version 1.4. https://CRAN.R-project.org/package=pedigree; 2013.

[53] Schwartz MK, Luikart G, Waples RS (2007). Genetic monitoring as a promising tool for conservation and management. Trends Ecol Evol.

[54] De Barba M, Waits LP, Garton EO, Genovesi P, Randi E, Mustoni A, Groff C (2010). The power of genetic monitoring for studying demography, ecology and genetics of a reintroduced brown bear population. Mol Ecol.

[55] Geffen E, Kam M, Hefner R, Hersteinsson P, Angerbjorn A, Dalen L, Fuglei E, Noren K, Adams JR, Vucetich J, Meier TJ, Mech LD, vonHoldt BM, Stahler DR, Wayne RK (2011). Kin encounter rate and inbreeding avoidance in canids. Mol Ecol.

[56] von Holdt BM, Stahler DR, Smith DW, Earl DA, Pollinger JP, Wayne RK (2008). The genealogy and genetic viability of reintroduced Yellowstone grey wolves. Mol Ecol.

[57] Åkesson M, Liberg O, Sand H, Wabakken P, Bensch S, Flagstad O (2016). Genetic rescue in a severely inbred wolf population. Mol Ecol.

[58] McNutt JW (1996). Sex-biased dispersal in African wild dogs, *Lycaon pictus*. Anim Behav..

[59] Bradley BJ, Doran-Sheehy DM, Lukas D, Boesch C, Vigilant L (2004). Dispersed male networks in western gorillas. Curr Biol.

[60] Marshall TC, Slate J, Kruuk LEB, Pemberton J (1998). Statistical confidence for likelihood-based paternity inference in natural populations. Mol Ecol.

[61] Kraus RHS, vonHoldt B, Cocchiararo B, Harms V, Bayerl H, Kuhn R, Forster DW, Fickel J, Roos C, Nowak C (2015). A single-nucleotide polymorphism-based approach for rapid and cost-effective genetic wolf monitoring in Europe based on noninvasively collected samples. Mol Ecol Res..

[62] Fabbri E, Caniglia R, Mucci N, Thomsen HP, Krag K, Pertoldi C, Loeschke V, Randi E (2012). Comparison of single nucleotide polymorphisms and microsatellites in non-invasive genetic monitoring of a wolf population. Arch Biol Sci.

