# Supplementary material for: Non-invasive genetic monitoring involving citizen science enables reconstruction of current pack dynamics in a re-establishing wolf population
Source: BMC Ecol. 2017 Dec 19;17:44. doi: 10.1186/s12898-017-0154-8 (PMC5738207; doi:10.1186/s12898-017-0154-8)
Supplement: Supplementary file 1 — Additional file 1. Additional details on methods and results are given in this supplementary file: a complete description of the molecular methods (Appendix 1); a full interpretation of pack dynamics (Appendix 2); details on microsatellite and primer sequences (Table S1); and the full pedigree outputted from the analysis pipeline and manual adjustments made to it (Table S2). [file 12898_2017_154_MOESM1_ESM.docx]

**Additional Information**

**Online Appendix 1 – details of genetic methods**

## *Genotyping*

Samples from a range of sources (tissue, faeces, hair, urine and secretion samples) were processed as a part of ongoing monitoring procedures over several years, and genotyping was conducted by several different researchers. Therefore, therefore the DNA extractions and genotyping protocols were modified during the course of the research roughly as follows: samples collected between 2013 and summer 2014 were processed using the *Initial protocol* (below), samples collected from late summer 2015 onwards were processed using the *Adjusted protocol* (below), and samples collected 7/2014 – 6/2015 were processed with different combinations of these two protocols during a protocol development phase.

In both protocols, genotyping was carried out a number of times on each sample, as recommended to ensure reliability of genotypes when analyzing very dilute DNA such as that extracted from feces [1, 2]. Each sample was first amplified in three independent replicates; at each locus, heterozygous genotypes were considered reliable if both alleles were observed in at least two replicates and homozygous genotypes if it was observed in all three [3, 4]. If any locus did not meet these requirements, a second series of three replicates was performed. If any locus yielded no reliable genotype, that locus was treated in the analysis as missing data.

*Initial protocol*

DNA was extracted using different commercially available kits depending on the DNA source material, following the manufacturer’s protocols with minor modifications. ***Faecal samples*** were extracted with a Qiagen QiAmp DNA Stool Mini kit, using about 200 mg taken from the frozen sample targeting the outer part of the fecal lobe where wolf intestinal cells are expected [5], and were eluted in 100 µl elution buffer. Tissue and secretion samples used the Qiagen QiAmp DNA Mini kit. ***Hair samples*** were digested in 300 µl ATL buffer with 20 µl Proteinase K and incubated on an orbital shaker at 56°C for 20 hours, with 160 µl of digested sample used for the rest of the extraction according to standard protocol. Final elution was done twice for 5 min in 50 µl AE buffer. S***ecretion samples*** were thawed to enable separation from the snow, then 200 µl of the melt liquid was digested using 200 µl ATL buffer and 20 µl proteinase K incubated for 10 min at 56°C. Subsequent steps followed standard protocol except that elution time was 5 min using 100 µl AE buffer. ***Muscle samples*** were digested in 250 µl ATL buffer with 20 µl proteinase K incubated at 56°C overnight on an orbital shaker. Elution used 100 µl AW buffer and elution time was 5 min. Negative controls were added in all extraction batches where samples were expected to have low quality or quantity DNA. The quantity and quality of all extracted DNA were quantified using a NanoDrop ND-1000 spectrophotometer.

All samples were genotyped at 17 established canid microsatellite loci (Supp. Table 1) that have been previously used in wolf population genetic studies, including in Finland [6–9). Separate amplification protocols were used for tissue and other sample types. Amplification was conducted in three multiplex PCRs, each using a 10 µl total reaction volume containing 3 µl template DNA, 1x Qiagen multiplex PCR master mix (QMP) and 0.1-0.4 uM of each primer (Supp. Table 1). All reactions used the same PCR program: initial denaturation at 95°C for 15 min, 40 amplification cycles of denaturation at 94°C for 30 s, annealing at 60°C for 90 s and extension at 72°C for 60 s, and a final extension step at 60°C for 30 min.

Individual genotypes were generated using fragment analysis of the fluorescently labeled PCR products. The allele sizes of the microsatellite markers were determined by capillary electrophoresis using ABI PRISM 3130xl Genetic Analyzer-sequencer (Applied Biosystems). PCR products were diluted into 80 µl MQ-water (1.5 µl of product from both panel 1 multiplexes; 1.0 µl from panel 2). Fragment analysis was carried out on 2 µl of diluted PCR product mixed with 10 µl of HiDi™ formamide and 0.1 µl of GS-600LIZ (ABI) size standard. The mixture was denatured for 3 min in +95°C before electrophoresis. The results of the fragment analysis were analyzed and individual genotypes were called using the genotyping program *GeneMarker* v.2.2.0 (Softgenetics, Inc.). After an automated initial allele call, all alleles were manually checked and confirmed before a final genotype was assigned.

*Adjusted protocol*

DNA was extracted using the Qiagen DNA Stool Mini kit for faecal samples as above, the Macherey Nagel NucleoSpin Tissue kit for swab, urine and secretion samples, and the NucleoSpin Tissue XS kit for hair samples. In all extractions the manufacturer’s protocols were followed with minor modifications. ***Tissue samples*** were extracted using the universal salt-extraction protocol of [10] with small modifications. ***Hair samples*** were digested in 200-400 ul T1 buffer with 20 µl Prot K, incubated in +56°C for 15 minutes, and extraction continued with standard protocol using 200-300 µl of digested sample. Final elution was in 20 µl BE buffer. ***Urine and secretion samples*** were thawed to enable separation from the snow, and 1.5 ml of the melt liquid was transferred into a new tube. The sample was centrifuged for 10 minutes at 13,000 x g and supernatant discarded. After this, the standard protocol was followed with a final elution volume of 60 µl BE buffer. One negative control was added in all extraction batches where the samples were expected to have low quality or quantity DNA.

All samples were genotyped at 17 microsatellite loci as above. Separate amplification protocols were used for tissue and other sample types. For ***tissue/swab samples with high quality DNA*** amplification was conducted in three multiplex PCRs, each using a 10 µl total reaction volume containing 1 µl template DNA, 1x Qiagen multiplex PCR master mix (QMP) and 0.1-0.4 uM of each primer (Supp. Table 1). All reactions used the same PCR program: initial denaturation at 95°C for 15 min, 35 amplification cycles of denaturation at 94°C for 30 s, annealing at 60°C for 90 s and extension at 72°C for 60 s, and a final extension step at 60°C for 10 min. For ***samples with degraded and/or low amounts DNA*** amplification was conducted in five multiplex PCRs, each using a 12 µl total reaction volume containing 3 µl template DNA, 1x Qiagen multiplex PCR master mix (QMP) and 0.1-0.3 uM of each primer (Supp. Table 1). All reactions used the same PCR program: initial denaturation at 95°C for 15 min, 40 amplification cycles of denaturation at 94°C for 30 s, annealing at 60°C for 90 s and extension at 72°C for 60 s, and a final extension step at 60°C for 30 min.

Individual genotypes were generated using fragment analysis of the fluorescently labelled PCR products. The allele sizes of the microsatellite markers were determined by capillary electrophoresis using ABI PRISM 3130xl Genetic Analyzer-sequencer (Applied Biosystems). PCR products of low quality DNA were diluted into 80 µl MQ-water as follows in panel 1: 3.0 µl + 2.0 µl + 1.0 µl + 2.0 µl of pcr product from multiplexes 1-3 and single pcr, respectively; 1.0 µl from panel 2. For high quality DNA 1.0 µl of product was diluted into 90 µl MQ-water from both panel 1 multiplexes; 1.5 µl from panel 2. Fragment analysis was carried out on 2 µl of diluted PCR product mixed with 10 µl of HiDi™ formamide and 0.1 µl of GS-600LIZ (ABI) size standard. The mixture was denatured for 3 min in +95°C before electrophoresis. The results of the fragment analysis were analyzed and individual genotypes were called using the genotyping program GeneMarker v.2.4.0 (Softgenetics, Inc.). After an automated initial allele call, all alleles were manually checked and confirmed before a final genotype was assigned.

**References**

[1] Morin PA, Chambers KE, Boesch C, Vigilant L. Quantitative polymerase chain reaction analysis of DNA from noninvasive samples for accurate microsatellite genotyping of wild chimpanzees (*Pan troglodytes verus*). Mol Ecol. 2001;10:1835–44.

[2] Taberlet P, Griffin S, Goossens B, Questiau S, Manceau V, Escaravage N, Waits LP, Bouvet J. Reliable genotyping of samples with very low DNA quantities using PCR. Nucl Acids Res. 1996;24:3189–94.

[3] Jansson E, Harmoinen J, Ruokonen M, Aspi J. Living on the edge: reconstructing the genetic history of the Finnish wolf population. BMC Evol Biol*.* 2014;14:64.

[4] Stansbury CR, Ausband DE, Zager P. A Long-Term Population Monitoring Approach for a Wide-Ranging Carnivore: Noninvasive Genetic Sampling of Gray Wolf Rendezvous Sites in Idaho, USA. J Wildl. Man. 2014;78:1040–49.

[5] Stenglein JL, Waits LP, Ausband DE, Zager P, Mack CM. Estimating gray wolf pack size and family relationships using noninvasive genetic sampling at rendezvous sites. J Mammal. 2011;92:784–95.

[6] Ostrander E.A., Sprague G.F. & Rine J. (1993) Identification and characterization of dinucleotide repeat (ca)n markers for genetic mapping in dog. Genomics **16**, 207–213.

[7] Fredholm M, Wintero AK. Variation of short tandem repeats within and between species belonging to the canidae family. Mamm Gen 1995;6:11–8.

[8] Francisco LV, Langston AA, Mellersh CS, Neal CL, Ostrander EA. A class of highly polymorphic tetranucleotide repeats for canine genetic mapping. Mamm Gen*.* 1996;7:359–62.

[9] Jansson E, Ruokonen M, Kojola I, Aspi J. Rise and fall of a wolf population: genetic diversity and structure during recovery, rapid expansion and drastic decline. Mol Ecol. 2012;21:5178–93.

[10] Aljanabi SM, Martinez I. Universal and rapid salt-extraction of high quality genomic DNA for PCR-based techniques. Nucl Acids Res. 1997;25:4692–3.

**Online Appendix 2 – details of pack dynamics interpreted from pedigree**

The earliest confirmed breeding pair in the data, AUL-001 and YLA-005 in Mynämäki, likely produced cubs every year from at least 2009, before our systematic monitoring began, to 2011 (high-confidence litters in 2010 and 2011, and likely litters also in 2008 and 2009). No further cubs were assigned to this pair after AUL-001 was shot in early 2012 aged 9, a high age for a wild wolf. YLA-005 remained in the record for a further two years, but with no evidence of continued reproduction. In 2012, their daughter YLA-002 inherited the breeding position and reproduced with a likely immigrant male, POY-006. In the following year 2013, neither these parents nor new cubs were detected in Mynämäki and POY-006 was identified as a breeder further east (Pöytyä; fig. 4), indicating that no breeding may have taken place in Mynämäki in 2013. In 2014, a new breeding pair was detected in Mynämäki consisting of the son of the previous breeding pair (YLA-003, resident) and the daughter of an established pair in another territory, Köyliö (YLA-004, disperser).

The first breeding individuals in Köyliö, KOY-008 and KOY-010 (both of unsampled or uncertain parents), appear in the data in 2013 when data collection in the area began. However, they are likely to have reproduced in 2011 or earlier: their daughter POY-008 was detected as a reproducer (min. 2 years old) in a neighbouring pack, Pöytyä, when sample collection began there in 2013. In 2015, the Köyliö male KOY-008 changed mate to produce cubs with his granddaughter, who had dispersed from the Pöytyä pack, giving the first instance of recent inbreeding in this population.

In the Pöytyä pack, we have evidence for reproduction by only one pair, a male dispersing as an adult after breeding in Mynämäki (POY-006) and a dispersing female born in the Köyliö pack (POY-008). The male’s movement makes it likely that this pack was established by this pair, effectively splitting the Mynämäki pack, but as the first detected breeding event in 2013 coincides with the beginning of intense monitoring in this area, the genetic data do not allow us to rule out earlier reproduction by other individuals in this territory.

The Köyliö pack also produced another pack founder, the female KOY-002 who dispersed to a new and (based on field observations) previously unoccupied territory in Raasepori, ~150km south-east of her natal pack. She and a likely immigrant male (TEN-001) were detected close together in this previously unoccupied area in winter/spring 2015, indicating a potential incipient breeding event. In summer 2015, a wildlife camera captured a young litter in the area, and indeed, our pedigree reconstruction including samples from 2015 confirmed that the newly-established pair had produced cubs that summer.

**Additional tables**

**Additional Table S1**. Details of the 17 microsatellites used for wolf genotyping and their amplification in the initial (samples collected up to summer 2015) and adjusted (samples collected from late summer 2015 onwards) protocols. The two panels contained of 14 dinucleotide repeat microsatellites *(CPH12, CPH8, AHT137, REN169O18, CPH4, CPH2, C20253, AHTk211, AHT121, AHTH130, C09173, CXX225, CXX279, INRA21)* and three tetranucleotide repeats *(C2001, C2096, C2088).* Forward primers were synthesized with a fluorescent dye in order to be detectable in the fragment analysis.

| **Initial protocol** | | |
| --- | --- | --- |
| **Panel 1** |  |  |
| **Primer** | **Multiplex and primer concentration for PCR(umol/l)** | **Primer sequence** |
| CPH12 | mp1 (0.4) | F:GGCATTACTTGGAGGGAGGAA |
|  |  | R:GTTTGATGATTCCTATGCTTCTTTGAG |
| CPH8 | mp1 (0.4) | F:AGGCTCACAATCCCTCTCATA |
|  |  | R:GTTTAGATTTGATACCTCCCTGAGTCC |
| C2001 | mp1 (0.4) | F:TCCTCCTCTTCTTTCCATTGG |
|  |  | R:GTTTGAACAGAGTTAAGGATAGACACG |
| AHT137 | mp1 (0.4) | F:TACAGAGCTCTTAACTGGGTCC |
|  |  | R:GTTTCCTTGCAAAGTGTCATTGCT |
| REN169O18 | mp1 (0.4) | F:CACCCAACCTGTCTGTTCCT |
|  |  | R:GTTTACTGTGTGAGCCAATCCCTT |
| CPH2 | mp2 (0.1) | F:TTCTGTTGTTATCGGCACCA |
|  |  | R:GTTTCTTGAGAACAGTGTCCTTCG |
| CPH4 | mp2 (0.4) | F:ACTGGAGATGAAAACTGAAGATTATA |
|  |  | R:GTTTACAGGGGAAAGCCTCATT |
| C2096 | mp2 (0.1) | F:CCGTCTAAGAGCCTCCCAG |
|  |  | R:GTTTGACAAGGTTTCCTGGTTCCA |
| AHTk211 | mp2 (0.2) | F:TTAGCAGCCGAGAAATACGC |
|  |  | R:GTTTATTCGCCCGACTTTGGCA |
| C2088 | mp2 (0.2) | F:CCTCTGCCTACATCTCTGC |
|  |  | R:GTTTAGGGCATGCATATAACCAGC |
| C20253 | mp2 (0.1) | F:AATGGCAGGATTTTCTTTTGC |
|  |  | R:GTTTATCTTTGGACGAATGGATAAGG |
| **Panel 2** |  |  |
| AHT121 | mp1 (0.1) | F:TATTGCGAATGTCACTGCTT |
|  |  | R:GTTTATAGATACACTCTCTCTCCG |
| AHTH130 | mp1 (0.2) | F:GTTTCTCTCCCTTCGGGTTC |
|  |  | R:GTTTGACGTGTGTTCACGCCAG |
| C09173 | mp1 (0.2) | F:ATCCAGGTCTGGAATACCCC |
|  |  | R:GTTTCCTTTGAATTAGCACTTGGC |
| CXX225 | mp1 (0.1) | F:AGCGACTATTATATGCCAGCG |
|  |  | R:GTTTCTCATTGGTGTAAAGTGGCG |
| CXX279 | mp1 (0.2) | F:TGCTCAATGAAATAAGCCAGG |
|  |  | R:GTTTGGCGACCTTCATTCTCTGAC |
| INRA21 | mp1 (0.1) | F:ATGTAGTTGAGATTTCTCCTACGG |
|  |  | R:GTTTTTAATGGCTGATTTATTTGGTGG |

| **Adjusted protocol** | | | | |
| --- | --- | --- | --- | --- |
| **Panel 1** |  |  |  |  |
| **Primer** | **Multiplexes and primer concentration for PCR (µM)** | | **Fluorescent label** | **Primer sequence** |
|  | High quality DNA | Low quality DNA |  |  |
| CPH12 | mp1 (0.4) | mp1 (0.2) | fam | F:GGCATTACTTGGAGGGAGGAA |
|  |  |  |  | R:GTTTGATGATTCCTATGCTTCTTTGAG |
| CPH8 | mp1 (0.4) | mp1 (0.2) | vic | F:AGGCTCACAATCCCTCTCATA |
|  |  |  |  | R:GTTTAGATTTGATACCTCCCTGAGTCC |
| C2001 | mp1 (0.4) | mp2 (0.1) | vic | F:TCCTCCTCTTCTTTCCATTGG |
|  |  |  |  | R:GTTTGAACAGAGTTAAGGATAGACACG |
| AHT137 | mp1 (0.4) | mp2 (0.2) | ned | F:TACAGAGCTCTTAACTGGGTCC |
|  |  |  |  | R:GTTTCCTTGCAAAGTGTCATTGCT |
| REN169O18 | mp1 (0.4) | single (0.2) | pet | F:CACCCAACCTGTCTGTTCCT |
|  |  |  |  | R:GTTTACTGTGTGAGCCAATCCCTT |
| CPH2 | mp2 (0.1) | mp3 (0.2) | fam | F:TTCTGTTGTTATCGGCACCA |
|  |  |  |  | R:GTTTCTTGAGAACAGTGTCCTTCG |
| CPH4 | mp2 (0.4) | mp1 (0.1) | fam | F:ACTGGAGATGAAAACTGAAGATTATA |
|  |  |  |  | R:GTTTACAGGGGAAAGCCTCATT |
| C2096 | mp2 (0.1) | mp3 (0.1) | ned | F:CCGTCTAAGAGCCTCCCAG |
|  |  |  |  | R:GTTTGACAAGGTTTCCTGGTTCCA |
| AHTk211 | mp2 (0.2) | mp3 (0.2) | pet | F:TTAGCAGCCGAGAAATACGC |
|  |  |  |  | R:GTTTATTCGCCCGACTTTGGCA |
| C2088 | mp2 (0.2) | mp2 (0.2) | pet | F:CCTCTGCCTACATCTCTGC |
|  |  |  |  | R:GTTTAGGGCATGCATATAACCAGC |
| C20253 | mp2 (0.1) | mp3 (0.1) | vic | F:AATGGCAGGATTTTCTTTTGC |
|  |  |  |  | R:GTTTATCTTTGGACGAATGGATAAGG |
| **Panel 2** |  |  |  |  |
| AHT121 | mp1 (0.1) | mp1 (0.1) | vic | F:TATTGCGAATGTCACTGCTT |
|  |  |  |  | R:GTTTATAGATACACTCTCTCTCCG |
| AHTH130 | mp1 (0.2) | mp1 (0.2) | fam | F:GTTTCTCTCCCTTCGGGTTC |
|  |  |  |  | R:GTTTGACGTGTGTTCACGCCAG |
| C09173 | mp1 (0.2) | mp1 (0.2) | pet | F:ATCCAGGTCTGGAATACCCC |
|  |  |  |  | R:GTTTCCTTTGAATTAGCACTTGGC |
| CXX225 | mp1 (0.1) | mp1 (0.1) | fam | F:AGCGACTATTATATGCCAGCG |
|  |  |  |  | R:GTTTCTCATTGGTGTAAAGTGGCG |
| CXX279 | mp1 (0.2) | mp1 (0.2) | ned | F:TGCTCAATGAAATAAGCCAGG |
|  |  |  |  | R:GTTTGGCGACCTTCATTCTCTGAC |
| INRA21 | mp1 (0.1) | mp1 (0.1) | ned | F:ATGTAGTTGAGATTTCTCCTACGG |
|  |  |  |  | R:GTTTTTAATGGCTGATTTATTTGGTGG |

**Additional Table S2**. Full output pedigree (main pedigree) with likelihoods of parental assignments and the final pedigree with manual adjustments to remove unrealistic assignments and confirm realistic low-confidence assignments. A parent ID of “us” means the most likely parent is unsampled, i.e. not one of the individuals in our dataset, and a parent ID of “-” shows where no confident and/or biologically likely assignment could be made. Offspring whose parentage assignments were manually changed are denoted with “a” for alterations or “c” for confirmations. Individuals are shown in alphabetical order.

| **Individual** | **Output pedigree** | | | | **Final (altered) pedigree** | | **Manual alteration or confirmation** |
| --- | --- | --- | --- | --- | --- | --- | --- |
|  | **Mother** | **Father** | **Mother likelihood** | **Father likelihood** | **Mother** | **Father** |  |
| AUL-001 | us | us | 0.1878 | 0.6627 | - | - |  |
| AUL-002 | AUL-001 | us | 0.507 | 0.9144 | AUL-001 | us | c |
| AUL-003 | AUL-001 | AUL-004 | 0.9576 | 1 | AUL-001 | - | a |
| AUL-004 | AUL-001 | us | 0.5655 | 0.9992 | AUL-001 | us | c |
| AUL-005 | AUL-001 | us | 0.5564 | 0.8267 | AUL-001 | us | c |
| AUL-006 | YLA-006 | us | 0.9627 | 0.9794 | - | us | a |
| AUL-007 | AUL-001 | Py1-Ind-010 | 0.9624 | 0.9562 | - | - | a |
| AUL-008 | AUL-002 | KOY-005 | 0.9533 | 0.2892 | AUL-002 | - |  |
| AUL-009 | AUL-002 | us | 0.7488 | 0.6863 | AUL-002 | - | c |
| AUL-010 | AUL-001 | YLA-005 | 1 | 1 | AUL-001 | YLA-005 |  |
| AUL-011 | AUL-001 | YLA-005 | 0.9657 | 0.9658 | AUL-001 | YLA-005 |  |
| AUL-012 | AUL-001 | YLA-005 | 0.8307 | 0.9993 | AUL-001 | YLA-005 |  |
| AUL-013 | AUL-001 | YLA-005 | 0.9999 | 0.9998 | AUL-001 | YLA-005 |  |
| AUL-014 | AUL-001 | YLA-005 | 1 | 1 | AUL-001 | YLA-005 |  |
| AUL-015 | Py1-Ind-002 | YLA-005 | 0.4198 | 0.9997 | AUL-001 | YLA-005 | a |
| KOY-001 | KOY-010 | KOY-008 | 0.9999 | 0.9995 | KOY-010 | KOY-008 |  |
| KOY-002 | KOY-010 | KOY-008 | 0.9912 | 0.9864 | KOY-010 | KOY-008 |  |
| KOY-003 | KOY-010 | KOY-008 | 0.984 | 0.9835 | KOY-010 | KOY-008 |  |
| KOY-004 | KOY-010 | KOY-008 | 1 | 1 | KOY-010 | KOY-008 |  |
| KOY-005 | KOY-010 | KOY-008 | 0.9999 | 0.9983 | KOY-010 | KOY-008 |  |
| KOY-006 | KOY-010 | KOY-008 | 1 | 1 | KOY-010 | KOY-008 |  |
| KOY-007 | KOY-010 | KOY-008 | 0.9978 | 0.9964 | KOY-010 | KOY-008 |  |
| KOY-008 | us | us | 0.97 | 0.9954 | us | us |  |
| KOY-009 | KOY-010 | KOY-008 | 1 | 1 | KOY-010 | KOY-008 |  |
| KOY-010 | AUL-001 | us | 0.4755 | 0.9976 | - | us |  |
| KOY-011 | KOY-010 | KOY-008 | 0.5818 | 0.9992 | KOY-010 | KOY-008 | c |
| Luke2015VS-KSS-Ind-001 | YLA-004 | YLA-003 | 0.5053 | 0.9988 | YLA-004 | YLA-003 | c |
| PAI-001 | POY-014 | POY-013 | 0.4807 | 0.6 | POY-008 | POY-006 | a |
| POY-001 | POY-008 | POY-006 | 0.9986 | 0.9859 | POY-008 | POY-006 |  |
| POY-002 | POY-008 | POY-006 | 0.9928 | 0.9089 | POY-008 | POY-006 |  |
| POY-003 | POY-008 | POY-006 | 0.9998 | 0.9938 | POY-008 | POY-006 |  |
| POY-004 | POY-008 | POY-006 | 0.9332 | 0.9202 | POY-008 | POY-006 |  |
| POY-005 | POY-008 | POY-006 | 0.9981 | 0.9874 | POY-008 | POY-006 |  |
| POY-006 | us | us | 0.7175 | 0.9134 | - | us |  |
| POY-007 | POY-008 | POY-006 | 0.9943 | 0.9802 | POY-008 | POY-006 |  |
| POY-008 | KOY-010 | KOY-008 | 0.9721 | 0.9952 | KOY-010 | KOY-008 |  |
| POY-009 | POY-014 | POY-001 | 0.661 | 0.6487 | POY-008 | POY-006 | a |
| POY-010 | POY-008 | POY-006 | 0.9999 | 0.9964 | POY-008 | POY-006 |  |
| POY-011 | POY-008 | POY-006 | 0.8437 | 0.5373 | POY-008 | POY-006 | c |
| POY-012 | POY-008 | POY-006 | 0.9963 | 0.9982 | POY-008 | POY-006 |  |
| POY-013 | POY-008 | POY-006 | 0.9958 | 0.9835 | POY-008 | POY-006 |  |
| POY-014 | POY-008 | POY-006 | 0.5071 | 0.4776 | POY-008 | POY-006 |  |
| POY-015 | Py1-Ind-007 | POY-001 | 0.8049 | 0.7559 | POY-008 | POY-006 | a |
| Py1-Ind-001 | YLA-004 | YLA-003 | 1 | 0.6697 | YLA-004 | YLA-003 | c |
| Py1-Ind-002 | YLA-004 | YLA-003 | 0.9999 | 0.9998 | YLA-004 | YLA-003 |  |
| Py1-Ind-003 | YLA-004 | YLA-003 | 0.4812 | 0.503 | YLA-004 | YLA-003 | c |
| Py1-Ind-004 | YLA-004 | YLA-003 | 0.9999 | 0.9995 | YLA-004 | YLA-003 |  |
| Py1-Ind-005 | YLA-004 | YLA-003 | 1 | 1 | YLA-004 | YLA-003 |  |
| Py1-Ind-006 | YLA-004 | YLA-003 | 0.9886 | 0.9967 | YLA-004 | YLA-003 |  |
| Py1-Ind-007 | POY-002 | Py1-Ind-009 | 0.7586 | 0.7587 | POY-008 | POY-006 | a |
| Py1-Ind-008 | POY-008 | POY-006 | 0.9751 | 0.9391 | POY-008 | POY-006 |  |
| Py1-Ind-009 | POY-008 | POY-006 | 0.9582 | 0.9583 | POY-008 | POY-006 |  |
| Py1-Ind-010 | KOY-007 | POY-001 | 0.6078 | 0.6146 | POY-008 | POY-006 | a |
| Py1-Ind-011 | POY-014 | Py1-Ind-008 | 0.7692 | 0.7719 | POY-008 | POY-006 | a |
| Py1-Ind-012 | POY-007 | KOY-008 | 0.9998 | 0.9998 | POY-007 | KOY-008 |  |
| Py1-Ind-013 | POY-007 | KOY-008 | 0.9998 | 0.9947 | POY-007 | KOY-008 |  |
| Py1-Ind-014 | POY-007 | KOY-008 | 1 | 1 | POY-007 | KOY-008 |  |
| Py1-Ind-015 | POY-007 | KOY-008 | 0.9999 | 1 | POY-007 | KOY-008 |  |
| Py1-Ind-016 | POY-007 | KOY-008 | 0.9988 | 0.9995 | POY-007 | KOY-008 |  |
| Py1-Ind-017 | POY-008 | POY-006 | 0.9993 | 0.9922 | POY-008 | POY-006 |  |
| REN-001 | us | SAL-001 | 0.9609 | 0.9033 | us | SAL-001 |  |
| SAL-001 | YLA-006 | us | 0.6244 | 0.997 | - | us | a |
| Te1-Ind-001 | KOY-002 | TEN-001 | 0.9754 | 1 | KOY-002 | TEN-001 |  |
| Te1-Ind-002 | KOY-002 | TEN-001 | 0.9999 | 1 | KOY-002 | TEN-001 |  |
| Te1-Ind-003 | Te1-Ind-006 | TEN-001 | 0.6725 | 0.9999 | KOY-002 | TEN-001 | a |
| Te1-Ind-005 | KOY-002 | TEN-001 | 0.9999 | 1 | KOY-002 | TEN-001 |  |
| Te1-Ind-006 | KOY-002 | TEN-001 | 1 | 0.991 | KOY-002 | TEN-001 |  |
| Te1-Ind-008 | KOY-002 | TEN-001 | 0.9997 | 0.9998 | KOY-002 | TEN-001 |  |
| TEN-001 | us | us | 0.9863 | 0.9984 | us | us |  |
| UmRK-Ind-001 | us | us | 0.9604 | 0.7831 | us | - |  |
| UmRK-Ind-002 | KOY-010 | KOY-008 | 0.6926 | 0.6924 | KOY-010 | KOY-008 | c |
| YLA-001 | Py1-Ind-004 | POY-006 | 0.7903 | 0.9999 | YLA-002 | POY-006 | a |
| YLA-002 | AUL-001 | YLA-005 | 0.9179 | 0.7638 | AUL-001 | YLA-005 | c |
| YLA-003 | YLA-002 | POY-006 | 0.9979 | 0.9964 | YLA-002 | POY-006 |  |
| YLA-004 | KOY-010 | KOY-008 | 0.9949 | 0.9909 | KOY-010 | KOY-008 |  |
| YLA-005 | us | us | 0.7373 | 0.9939 | - | us |  |
| YLA-006 | YLA-004 | YLA-003 | 0.9999 | 0.9999 | YLA-004 | YLA-003 |  |
| YLA-007 | YLA-004 | YLA-003 | 0.6688 | 0.9958 | YLA-004 | YLA-003 | c |
| YLA-008 | POY-011 | YLA-009 | 0.7529 | 0.799 | YLA-004 | YLA-003 | a |
| YLA-009 | YLA-004 | YLA-003 | 0.6615 | 0.9967 | YLA-004 | YLA-003 | c |
| YLA-010 | YLA-002 | POY-006 | 0.9993 | 0.9998 | YLA-002 | POY-006 |  |
